# Bayesian integrated modeling of expression data: a case study on RhoG

**DOI:** 10.1186/1471-2105-11-295

**Published:** 2010-06-01

**Authors:** Rashi Gupta, Dario Greco, Petri Auvinen, Elja Arjas

**Affiliations:** 1 Department of Mathematics and Statistics, University of Helsinki, P.O. Box 68, FIN-00014, Helsinki, Finland; 2 Institute of Biotechnology, University of Helsinki, P.O. Box 56, FIN-00014, Helsinki, Finland; 3 National Institute for Health and Welfare (THL), Mannerheimintie 166, 00300 Helsinki, Finland

## Abstract

**Background:**

DNA microarrays provide an efficient method for measuring activity of genes in parallel and even covering all the known transcripts of an organism on a single array. This has to be balanced against that analyzing data emerging from microarrays involves several consecutive steps, and each of them is a potential source of errors. Errors tend to accumulate when moving from the lower level towards the higher level analyses because of the sequential nature. Eliminating such errors does not seem feasible without completely changing the technologies, but one should nevertheless try to meet the goal of being able to realistically assess degree of the uncertainties that are involved when drawing the final conclusions from such analyses.

**Results:**

We present a Bayesian hierarchical model for finding differentially expressed genes between two experimental conditions, proposing an integrated statistical approach where correcting signal saturation, systematic array effects, dye effects, and finding differentially expressed genes, are all modeled jointly. The integration allows all these components, and also the associated errors, to be considered simultaneously. The inference is based on full posterior distribution of gene expression indices and on quantities derived from them rather than on point estimates. The model was applied and tested on two different datasets.

**Conclusions:**

The method presents a way of integrating various steps of microarray analysis into a single joint analysis, and thereby enables extracting information on differential expression in a manner, which properly accounts for various sources of potential error in the process.

## Background

Microarrays are popular high-throughput biological assays that measure the expression level of thousands of genes in the biological samples and generate large, complex datasets. In spite of the advances in technology, it is a major challenge to produce reliable gene expression data with a high signal-to-noise ratio, and analyze these large datasets in an adequate manner. Analyzing microarray data is usually performed in a step-wise manner, starting with, (i) normalization of the intensity measurements, to adjust or account for systematic technical variation, (ii) correcting dye-bias if dye-bias remains after normalization, (iii) identifying differentially expressed genes on the normalized data, and completing the analysis with (iv) functional annotation of the differentially expressed genes. All these steps are regarded as independent, but they are crucial for any biologically meaningful analysis.

Normalization is an integral part of the analysis, aiming at retaining the systematic effects resulting from the biological process of interest while removing the systematic technical variations occurring due to experimental variability. Normalization has researched for quite some time and publications proposing new procedures are available [1-4]. Some datasets display a consistent bias for a given probe in either Cy3 or Cy5 direction even after the data have been normalized using median-centered and lowess normalization methods. This bias is called dye bias and it is observed on a variety of platforms and labeling systems, including PCR-spotted and short oligonucleotide labeling methods. Many experimentalists and statisticians recommend using a dye-swap design to correct for this bias. Some publications have shown by considering experimental data that, if uncorrected, this bias can lead to the erroneous identification of genes [5-7].

Identification of differentially expressed genes is usually the main goal of microarray experiment. Chen *et al*. [8] assessed differentially expressed genes by calculating fold changes between genes under different conditions. Fold-change method, the simplest and the most intuitive method for finding genes that are differentially expressed, has many drawbacks. Later, improved methods based on t-test, regularized t-test [9,10] were proposed. Model based approaches have also been published to identify differentially expressed genes. Most methods listed in the literature use point estimates of expression and depend upon replicates available for the estimation of variances.

Step-wise analysis of the microarray data has two major drawbacks: (i) output from one step acts as direct input to the next, without attempting to account for the uncertainties associated with the value that was obtained; as a consequence, (ii) re-analyzing the data by altering the method used for a single step will often produce conflicting results. For this reason, Bhattacharjee *et al*. [11] proposed a method that aims at integrating the independent steps, so that uncertainties from each step could be accounted systematically. Lewin *et al*. [12] also proposed an integration of the normalization and classification step by using a Hierarchical Bayesian model. These proposed integrated approaches performed better than their step-wise approach counterparts. Moreover, the Bayesian formulation enables a much richer output than current step-wise analyses.

In here, we also propose an integrated statistical model under the Bayesian framework, where normalization and differential expression are modeled jointly, and correction of the saturated signal is also incorporated. Saturation refers to the optical saturation and not chemical saturation. Such (optical) signal saturation occurs in the scanning of hybridized arrays when the digitalized signal from a pixel exceeds the scanner's upper threshold of detection (2^16^-1 = 65535, for a 16 bit computer storage system). Saturation causes a downward bias in gene expression measurements, which then affects high level analysis, such as class prediction, class comparison or clustering that utilizes these signals [13].

Usually, data extracted from a single scan and a single scanner setting is used for all high level analyses. However, a single setting is unable to capture correctly the expression of both weakly and highly expressed genes. As a result, the sensitivity level of the scanner is adjusted to get reliable measurements from all fluorescent spots present on the hybridized array. Scanner sensitivity has to be raised to a certain level to ensure that the signal from weakly expressed genes exceeds the intrinsic noise level of the scanner, but this causes saturation for highly expressed genes. Several methods [14-19] have been proposed for correcting the bias caused by signal saturation.

In here, we extend our previous work (Gupta *et al*. [19]) on handling signal saturation by using several scans at varying scanner sensitivities. We propose an integrated statistical approach where correcting signal saturation, systematic array effects, gene-specific dye effects, and differential expression are modeled simultaneously. We estimate our model in a fully Bayesian way with the WinBUGS software [20]. The Bayesian framework allows for joint estimation of a large number of parameters, and enables us to obtain here the posterior distribution of any parameter in the model and of any function of such parameters. We show how to exploit these posterior distributions to assess differential expression, using multiple criteria for this purpose. The uncertainties in the parameter estimates are thereby incorporated in a natural manner into a proposed list of candidate genes.

## Method

### Data

RhoG is a protein belonging to the family of the small GTPases [21,22]. It is involved in several intracellular signaling pathways regulating cell motility and adhesion to extracellular matrix. Together with Cdc42 and Rac1, RhoG is able to elicit formation of both filopodia and lamellipodia. Neurite formation and regulation of axon dynamics in neurons are more specific functions in which RhoG is acting together with other Rho proteins and their interactors. Within the cells, Rho proteins can be found in an active form and inactive form. Mutants of RhoG (RhoG12 and RhoG17) can be used to keep the protein in a constitutively activated (mutation of the 12^th ^amino acid) or inactivated (mutation of the 17^th ^amino acid) form. In this study we investigate effect of mutants RhoG12 and RhoG17 on the gene expression of HeLa cell lines.

### Dataset-1

The DNA microarrays used for studying the effect of RhoG17 in HeLa cells were Agilent human 4 × 44 k and contained about 44000 60-mer oligonucleotide probes. Three replicate arrays were made initially but only two were used due to some technical problem in one of the arrays. Each array was scanned three times using Axon GenePix 4200AL scanner by varying the photomultiplier tube (PMT). The design of the experiment along with the configuration of PMT used to make multiple scans is given in Table 1. The dataset-1 is available as Additional file-1.

**Table 1:** Design details along with the configuration of PMT used to obtain multiple scans for two replicate arrays of dataset-1.

Dye		Array 1	Array 2
		RhoG17	RhoG17
**Cy3**	Scan-1 (PMT)	460	460
	Scan-2 (PMT)	410	410
	Scan-3 (PMT)	360	360
		Control	Control
**Cy5**	Scan-1 (PMT)	680	680
	Scan-2 (PMT)	630	630
	Scan-3 (PMT)	580	580

### Dataset-2

The DNA microarrays used for studying the effect of RhoG12 in HeLa cells were produced by the Turku Center for Biotechnology, University of Turku, Finland and contained 16,000 human cDNAs spotted in duplicate. Three arrays comparing wild type HeLa cells with RhoG12 mutant were prepared. One of the replicate arrays had the labeling orientation of the sample reversed. Each array was scanned three times using ScanArray 5000 scanner by varying the laser power. Table 2 shows design of the experiment along with configuration of photomultiplier tube (PMT) and laser power (LP) used to make multiple scans. The dataset-2 is available as Additional file-2.

**Table 2:** Design details along with the combinations of PMT and LP used to obtain multiple scans for three replicate arrays of dataset-2.

Dye		Array 1	Array 2	Array 3
		Control	Control	RhoG12
**Cy3**	**PMT Gain**	80	85	80
	Scan-1 (LP)	90	100	90
	Scan-2 (LP)	80	90	80
	Scan-3 (LP)	70	80	70
		RhoG12	RhoG12	Control
**Cy5**	**PMT Gain**	90	98	90
	Scan-1 (LP)	100	100	100
	Scan-2 (LP)	90	90	90
	Scan-3 (LP)	80	80	80

For details about RNA extraction, probes labeling, and microarray hybridization for the two datasets, see Additional file-3.

### Bayesian hierarchical model

The model aims at finding differentially expressed genes under *c*_*max *_conditions (here *c*_*max *_= 2, experimental and control, but *c*_*max *_can be more than two, for example, when comparing multiple conditions over time), each replicated on *r*_*max *_arrays (here *r*_*max *_= 2 (for dataset-1) and 3(for dataset-2)), and each array scanned *s *times (here *s*_*max *_= 3) under different scanner settings. We assume that, under condition *c*, each gene *i *has an underlying signal, which cannot be measured directly. We call this signal the *true latent intensity *of the gene under condition *c *and denote it by *T*_*ic*_, *c *= 1, 2; *i *= 1, 2, ..., *N*, where *N *is the number of spots used in the experiment. The entire model is defined on the logarithmic scale, base e.

Signal correction is done separately for each replicate by combining three scans made by varying the scanner settings for that replicate. Let *Q*_*icr *_represent latent intensity of gene *i *under condition *c *on replicate *r*. The scanner settings used in the first scan for each replicate are chosen to correspond to the situation, where only a single scan would be made; therefore these first scans form a natural basis for calibrating the latent intensities *Q*_*icr*_. They are also expected to capture, without a downward bias caused by saturation, spots that do not have abundant levels of RNA. The second and the third scans were made by choosing the scanner settings so that their measured signals would be weaker. Latent intensities corresponding to the second and third scans are now assumed to be linked to *Q*_*icr *_by simple functional relationships, respectively by *f*_*cr*2_(*Q*_*icr*_) and *f*_*cr*3_(*Q*_*icr*_) (discussed briefly later).

Let *Y*_*icrs *_denote the observed intensity for spot *i *under condition *c *and scan *s *of replicate *r*. As discussed in Gupta *et al*. [19], the relation between the observed and the latent intensity is non-linear. If there were no measurement errors, we could write the observed intensity *Y*_*icrs *_in the form *Y*_*icrs *_= *f*_*crs*_(*Q*_*icr*_). However, extraction of intensities of genes from scanned microarrays always involves some measurement errors. Here we assume that the errors are modulated by the latent signal level in a log-additive fashion. More exactly, we assume that for the observed intensities, which are below a certain threshold so that saturation has no effect, the relationship between observed and latent intensities can be expressed as:(1)

where, *ε*_*icrs *_is the error associated with spot *i *under condition *c *and scan *s *of replicate *r*. We further assume that the estimated latent intensity *Q*_*icr *_of gene *i *under condition *c *on replicate *r *can be modeled with additive gene, array and dye effects:(2)

where, *T*_*ic *_is the *true latent intensity *of a gene *i *under condition *c*, *A*_*ir *_is the array effect, and *β*_*i *_is the gene-specific dye effect. Since for cDNA experiments both the control and the experimental samples are hybridized on the same array, the array effect (*A*_*ir*_) is not dependent on the condition *c*. The gene-specific dye-bias correction (*β*_*i*_) is only applied when the values are taken from Cy5 intensity data, as enforced by the indicator function *I*(Cy5)*_cr. _*However, the symmetric model in which the correction is applied to Cy3 channel only would perform identically with the difference that the bias terms would be negated. A similar gene-specific dye bias correction was used in Kelley *et al*. [7].

The functions *f*_*cr2 *_and *f*_*cr3 *_in equation (1) are unknown and need to be estimated from the data. We assume these functions to be increasing and continuous. For their estimation, we decided to break the whole range of gene expression data (log_e_(200), log_e_(65535)) into small intervals yet ensuring enough data points in each of these intervals. We call these intervals as *I*_1_, *I*_2_, ... *I*_k_, and assume a simple linear form for *f*_*cr2 *_and *f*_*cr3 *_in each interval. In other words, we set(3)

where, L(*I*_*k*_) is the length of the k*^th ^*interval. The array effects (*A*_*ir*_) are estimated over the set of intervals *I*_1_, *I*_2_, ... *I*_*k*_, subject to the constraints ∑_*r *_*A*_*jr *_= 0, *j *= 1, 2, ...., *k *to ensure identifiability. Estimation of array effects over a set of intervals is similar to the intensity based estimation of array effects previously reported in Yang *et al*. [1] and Dudoit *et al*. [4].

To complete the specification of the model, we assumed Uniform prior distribution over the interval [0, 15] on logarithmic scale for *T*_*ic*_. The array effects *A*_*jr *_were assigned Normal priors with mean 0 and precision 0.1 (inverse of variance). The parameters *b*_*jcr *_and *d*_*jcr *_were assigned Uniform priors over the interval [0, 5]. Gene specific dye effects *β*_*i *_were also assigned Normal priors with mean 0 and precision 0.1. The errors *ε*_*icrs *_are assumed to be independent and identically distributed Normal random variables with mean 0 and interval dependent variances *η*^2^_*jcrs*_, where *s *= 1, 2, 3; *j *= 1, 2, ...., *k*. The interval dependent precision parameters (*η*_*jcr1*_^2^, *η*_*jcr2*_^2^, and *η*_*jcr3*_^2^; *j *= 1, 2, ..., *k*) were assigned gamma priors with parameters (0.001, 0.001).

Finally, as per Gupta *et al*. [19], to account for the effect of saturation, we treated signal measurements exceeding the threshold of log_e_(45000) as 'missing data'. We compensated for the resulting loss of information by applying model-based data augmentation and using the measurements taken from the second and/or the third scan which had been obtained by varying scanner settings.

### Implementation

The model was formulated in BUGS language and parameter estimation was performed using WinBUGS [20].

### Rules for selecting genes

Using the Bayesian model as specified above and with the available data, we can estimate, for each gene *i, i *= 1,....., *N*, the joint posterior distribution of (*T*_*i*1_, *T*_*i*2_), *i.e*., of the true underlying expression levels for the two conditions involved. Based on this, we can further determine the posterior distribution of *D*_*i *_= *T*_*i*_1 -*T*_*i*2_, *i *= 1,....., *N*, which represent the differential expression between conditions 1 and 2 in gene *i*. There are several ways in which the posterior distribution of *D*_*i *_can be exploited with the aim of identifying differential expression. Here we propose a method where we first select suitable threshold values *D*_*thres*_^+ ^and *D*_*thres*_^- ^for such differences and then consider a ranking based on the posterior probabilities:(4)

Genes are selected as being potentially up-regulated if *p*_*i*_^+ ^> *p*_*cut *_and down-regulated if *p*_*i*_^- ^> *p*_*cut*_, where again the cut-off point *p*_*cut *_needs to be chosen in advance. These posterior probabilities (*p*_*i*_^+ ^and *p*_*i*_^-^) are easily estimated by counting the proportion of MCMC samples in which the chosen criteria are satisfied. The choice of the controlling threshold values *p*_*cut*_, *D*_*thres*_^+ ^and *D*_*thres*_^- ^depends on the biological question being studied, and can be problematic to choose. However, in practice, the values are chosen only after a preliminary analysis of the data.

The above-mentioned criterion is quite similar to the criterion used in Lewin *et al*. [12], for selecting interesting genes. Other criteria for ranking genes include the use of standardized differences, *z*_*i *_= mean(*D*_*i*_)/sd(*D*_*i*_), and determining the highest percentile for which the credibility interval for *D*_*i *_does not cover zero [23]. It is important to note that identification of differentially expressed genes is here based directly on determining the gene-wise posterior probabilities that the latent 'true' difference in expression in the two conditions exceeds a certain threshold. Thus our method does not use the general framework of statistical hypothesis testing, involving, for example, *p*-values, or corrections of significance levels to account for multiple testing. Unlike Lewin *et al*. [12], we also have here not made an attempt to calibrate the chosen thresholds on the basis of frequentist criteria such as False Discovery/Non-Discovery Rate.

## Results and Discussion

### Application to dataset-1

The model under "Bayesian hierarchical model" without parameter (*β*_*i*_) was applied to dataset-1 to illustrate the criterion presented under "Rules for selecting genes". Since both replicate arrays from dataset-1 have the same dye-orientation, the dye-bias in the data cannot be assessed.

### Computational details and parameter estimation

For dataset-1, foreground median values for each condition without background correction were used for the analysis. As a result, we had no negative values. This particular dataset had 43,376 genes (on single array) × 2 (replicates used) × 3 (scans used) × 2 (dyes/conditions) = 520,512 data points to be used for the analysis. The current model runs in OpenBUGS version 2.01 on Intel Pentium processor 2.80 GHz with 1 GB RAM and takes approximately 4 seconds per iteration using two chains in parallel. Convergence was monitored visually (*i.e*. by the mixing of two chains) and two chains of 10,000 iterations each were generated to check the convergence of the parameter estimates under consideration. Thereafter a sample of size 10,000 was generated to make inference.

Owing to the intensity based structure and for computational convenience, the entire range of gene expression was divided into four intervals: *I*_1 _= (log_e_(200), log_e_(2000)), *I*_2 _= (log_e_(2000), log_e_(5000)), *I*_3 _= (log_e_(5000), log_e_(11000)), *I*_4 _= (log_e_(11000), -). This division was based on the measurement reading from scan-1. The posterior median estimates of the parameters (*b*_*jcr*_, *d*_*jcr*_) over the two conditions and for a single replicate are summarized in Table 3. The estimates are not the same over the four intervals in any of the two replicates, thus providing evidence of the intensity dependent structure of our data. The array effects (*A*_*jr*_) were also estimated over the same intervals, subject to the constraints ∑_*r *_*A*_*jr *_= 0 to ensure identifiability. The array effects (in terms of posterior median and sd) over the two replicates are shown in Table 4.

**Table 3:** Posterior median estimates of the parameters (*b*, *d*) for two conditions over replicate-1 of dataset-1.

Intensity-range		Posterior median estimate of *b*, *d *(median ± sd) for the two condition over replicate-1			
**Lower limit**	**Upper limit**	**Condition 1**		**Condition 2**	
		***b***	***d***	***b***	***d***
log_e_(200)	log_e_(2000)	0.9158 ± 0.0001	0.8370 ± 0.0001	0.9009 ± 0.0001	0.7952 ± 0.0001
log_e_(2001)	log_e_(5000)	0.9084 ± 0.0003	0.8275 ± 0.0003	0.9230 ± 0.0003	0.8325 ± 0.0003
log_e_(5001)	log_e_(11000)	0.9116 ± 0.0005	0.8354 ± 0.0005	0.9344 ± 0.0005	0.8554 ± 0.0005
log_e_(11001)	-	0.9206 ± 0.0006	0.8554 ± 0.0006	0.9436 ± 0.0006	0.8798 ± 0.0006

**Table 4:** Posterior median estimates of the array effect over the four intervals and over two replicates of dataset-1.

Intensity range		Posterior median estimate of array effect (median ± sd) over replicates	
**Lower Limit**	**Upper Limit**	**Replicate 1**	**Replicate 2**
log_e_(200)	log_e_(2000)	0.0018 ± 0.0006	-0.0094 ± 0.0006
log_e_(2001)	log_e_(5000)	-0.3107 ± 0.0039	0.3143 ± 0.0039
log_e_(5001)	log_e_(11000)	-0.3288 ± 0.0061	0.3302 ± 0.0061
log_e_(11001)	-	-0.2883 ± 0.0049	0.2910 ± 0.0049

The breakpoints were selected using visual inspection, but it would also be possible to treat them as model parameters and then estimate them jointly with *b*_*jcr*_, *d*_*jcr *_and *A*_*jr*_. This was not done here because of the additional computational burden that would have resulted in analyzing the huge dataset.

### Discussion of decision rules

**Figure 1:**
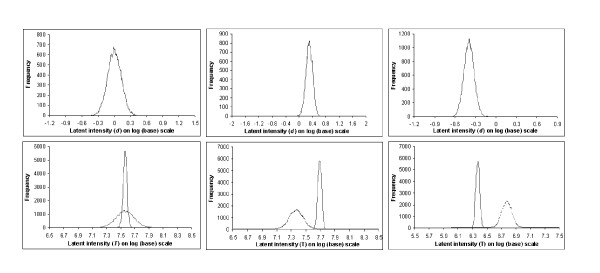
**Plot of posterior distribution of *D*_*i *_= *T*_*i*1_-*T*_*i*2 _for three genes**. In the upper panel, posterior distributions of the difference *D*_*i *_= *T*_*i*1_-*T*_*i*2 _are shown for three genes of dataset-1: a non-differentially expressed gene (left), an up-regulated gene (center), and a down-regulated gene (right). In the lower panel, the corresponding posterior distributions are shown for the latent variable *T*_*i*1 _corresponding to the experimental condition (solid line), and for *T*_*i*2 _corresponding to the control (dotted line).

As discussed before, the posterior distribution of the parameter *D*_*i *_= *T*_*i*1_-*T*_2 _represents the differential expression between conditions 1 and 2 in a gene. The uncertainty in its estimation is reflected in the shape of its distribution. A highly consistent response leads to a tighter posterior distribution, and a less consistent pattern will result in a flatter (sometimes multi-modal) posterior distribution. Genes that are not differentially expressed have their posterior distribution centered around zero. This can be seen in Figure 1 (upper panel, left) for a non-differentially expressed gene. Similar posterior distributions are shown for an up-regulated gene (upper panel, center) and a down-regulated gene (upper panel, right). The corresponding posterior distributions of the latent variables (*T*_*ic*_) under the two conditions leading to the estimation of the posterior distribution of the difference (*D*_*i*_) are also shown in Figure 1 (lower panel).

**Figure 2:**
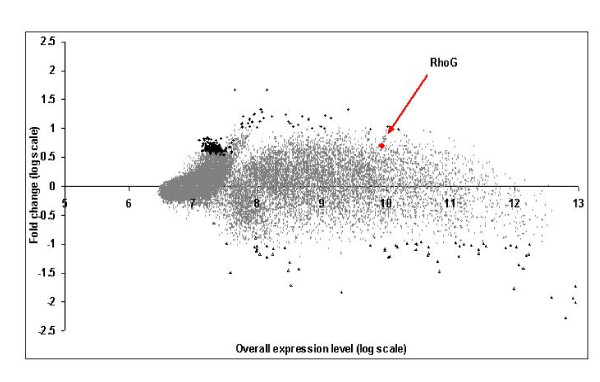
**Plot of point estimates (posterior means) of log-fold change *D*_*i *_against the overall expression (*T*_*i*1 _+ *T*_*i*2_)/2 for dataset-1**. Genes with *p*_*i*_^+ ^≥ 0.99 are plotted with diamonds and those with *p*_*i*_^- ^≥ 0.99 are plotted with triangles. The gene RhoG with *p*_*i*_^+ ^= 0.91 is plotted with a red circle.

Figure 2 shows point estimates (posterior means) of log-fold change *D*_*i *_versus overall expression (*T*_*i*1 _+ *T*_*i*2_)/2. We declared here genes as up-regulated if *p*_*i*_^+ ^> *p*_*cut *_and down-regulated if *p*_*i*_^- ^> *p*_*cut*_, with *p*_*cut *_= 0.99 and *D*_*thres*_^+ ^= *D*_*thres*_^- ^= 0.3 (on log scale). 270 genes came up as differentially expressed using these threshold values, 212 with *p*_*i*_^+ ^> 0.99 and 58 with *p*_*i*_^- ^> 0.99. The gene RhoG, which was expected to be up-regulated in this experiment, is also marked in Figure 1. It was identified with *p*_*i*_^+ ^= 0.91 and with a fold change of + 1.99 (on the natural scale). The up-regulation of both transgene and endogenous RhoG was validated (see Additional file 1, qPCR results) and suggests that there might be mechanisms by which RhoG regulates its own expression.

**Figure 3:**
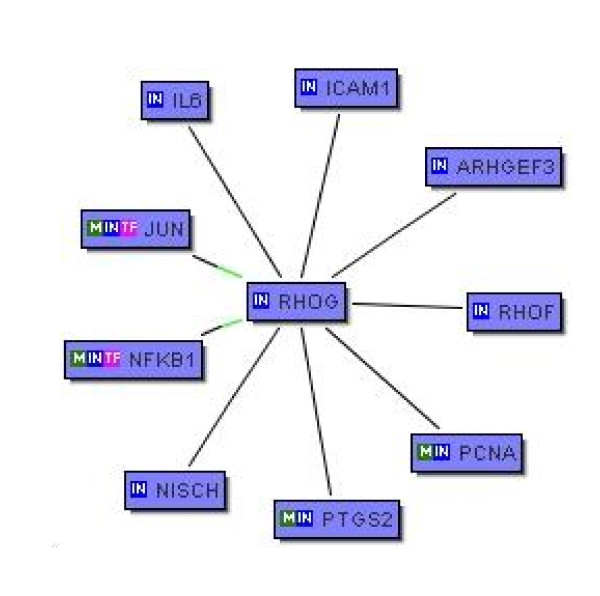
**A pictorial representation of the relation of nine genes co-cited with RhoG**. The blue boxes (nodes) represent the genes. The "black" edges indicate co-citation of two genes in the PubMed database; the "green" edges indicate a possible regulatory role of JUN and NFKB1 on the expression of RhoG.

Among the 270 genes, we searched for RhoG- related genes in Pubmed literature database using the software Bibliosphere http://www.genomatix.de/products/BiblioSphere/. Among the list of candidate genes, nine genes were identified as being co-cited with RhoG. A pictorial representation of the relation of these nine genes is shown in Figure 3. The black edges depict co-citation of the two genes and green edges indicate possible regulatory roles of JUN and NFKB1. Table 5 presents the estimated fold change of these 9 genes along with brief comments, their estimated posterior probabilities and Pubmed Id (PMID).

**Table 5:** Brief description and comments on some genes (of datset-1) found to be differentially expressed and associated with RhoG from literature.

Gene	Comment	Fold change(natural scale)	Pubmed Id (PMID)	Posterior probabilities
ARHGEF3	ARHGEF3 form complex with G proteins and stimulate Rho-dependent signals.	2.2	12221096	p^+ ^= 1
ICAM1	ICAM1 binds to integrins of type CD11a/CD18, or CD11b/CD18 and stimulates intercellular signaling.	1.6	17875742	p^+ ^= 0.9913
IL6	IL6 is an immunoregulatory cytokine that activates a cell surface signaling assembly composed of IL6, IL6RA, and the shared signaling receptor gp130.	4.2	15578470	p^+ ^= 1
JUN	This gene encodes a protein which interacts directly with specific target DNA sequences to regulate gene expression.	1.8	12739001, 1620121, 9671479, 10744696	p^+ ^= 0.9935
NFKB1	NFKB is a transcription regulator that is activated by various intra-and extra-cellular stimuli. Activated NFKB translocates into the nucleus and stimulates the expression of genes involved in a wide variety of biological functions.	1.9	12670394, 11803464, 12376551	p^+ ^= 0.9942
NISCH	NISCH is involved in the regulation of cell migration and cell invasion.	1.9	12890925	p^+ ^= 0.9965
PCNA	PCNA is found in the nucleus and is a cofactor of DNA polymerase delta. The encoded protein helps increase the processivity of leading strand synthesis during DNA replication.	0.76	12167123	p^- ^= 1
PTGS2	Prostaglandin-endoperoxide synthase is the key enzyme in prostaglandin biosynthesis, and acts both as a dioxygenase and as a peroxidase.	2.3	10974444	p^+ ^= 1
RHOF	RHOF functions cooperatively with CDC42 and Rac to generate filopodia increasing the diversity of actin-based morphology.	3.7	15894457	p^+ ^= 0.9994

### Gene ontology categories enriched among the differentially expressed genes

Our aim was to identify the GO terms that were enriched among the 270 genes identified as differentially expressed using DAVID annotation tool [24]. Several categories were over represented with Fisher's exact test p-value 0.05 but we present in Table 6 a few selected categories that contain novel genes that might be functionally related to RhoG based on published data. The list of GO terms associated with the differentially expressed genes is available as Additional file 4.

**Table 6:** Some selected GO categories, along with the numbers of varying and analyzed genes from dataset-1.

Gene ontology categories	Number of genes estimated as varying	Number of genes analyzed
GTPase activity	6	212
Endosome transport	3	41
developmental process	46	3262
cell proliferation	15	796
cell cycle	16	894
vesicle-mediated transport	13	509
endocytosis	8	197
cell differentiation	32	1835
Cellular component organization and biogenesis	48	2723
Organelle organization and biogenesis	22	1195
Establishment and/or maintainence of chromatin architecture	9	315

Regulation of actin cytoskeleton dynamics is one of the central effects of RhoG on cells. RhoF (or Rif) was one of the genes that showed up in this category [25]. RhoF is involved in the filopodia formation through mDia2. Among the small GTPases, RhoA is a key regulator of actin cytoskeleton. Presently, little is known about the possible functional relationships of RhoA and RhoG. However, we identified several interesting candidate genes that could participate in the possible cross-talk between these Rho proteins: ROCK2 is a classical RhoA-linked regulator of actin [26] and two RhoA GEFs (ARHGEF10L [27] and ARHGEF3 [28]) exhibit ways in which RhoG could regulate the activity of RhoA by inducing the expression of their regulators.

We also identified Cdc42 regulators (Chiamerin, see Additional file 1, qPCR results) indicating that there are unknown cross-talk between RhoG and other RhoGTPases in regulating actin cytoskeleton homeostasis. Moreover, ARPC3, a part of the Arp2/3 complex, was identified [29,30]. This complex is one of the actin nucleation apparatuses responsible for many actin-related functions like endosytosis, lamellipodia formation and filopodia formation. Our list of candidate genes helps us understand how regulatory genes like RhoG are performing their multitasking in cell dynamics.

### Step-wise analysis using existing approaches

For a comparison, dataset-1 was also analyzed in a step-wise manner using the existing popular softwares/procedures. The data from the multiple scans of each replicate and from the two dyes were first combined using the multiscan package in R. The multiscan package implements the method of Khondoker *et al*. [17], for estimating gene expressions from multiple laser scans of hybridized microarrays. The method proposed in Khondoker *et al*. [17] has already been compared with a similar method from Gupta *et al*. [19] which was utilized in this paper for estimating gene signals from multiple scans. Gupta *et al*. [19] also showed that the estimated gene signal from multiple scans gave better results when utilized for high level analysis than the gene signal data from a single scan.

The combined signals from the multiple scans of the three replicates and for the two dyes were normalized using Quantile normalization method in R [31]. Limma was used to fit a model and to identify differentially expressed genes. We used DAVID [24] for the functional annotation of the selected genes. This step-wise analysis identified three broad functionalities "cell differentiation", "cell cycle" and "developmental process" (also listed in Table 6, results from integrated approach) but failed to identify other specific functionalities associated with the experiment.

### Assessing dye bias

**Figure 4:**
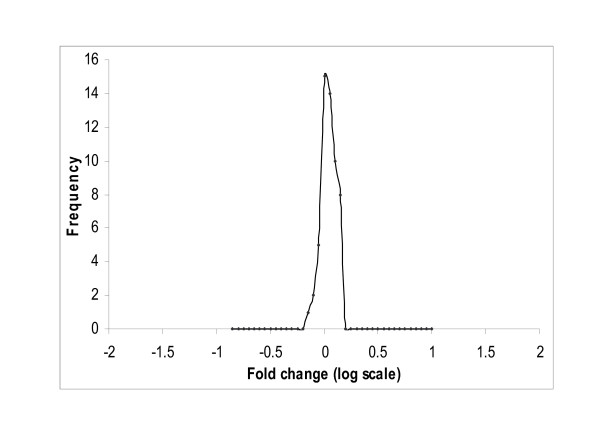
**Histograms of point estimates (median of posterior distribution) of *D*_*i *_for GAPDH**. These point estimates are of the 56 replicates (on the same array) for a house keeping genes (GAPDH) of dataset-2.

**Figure 5:**
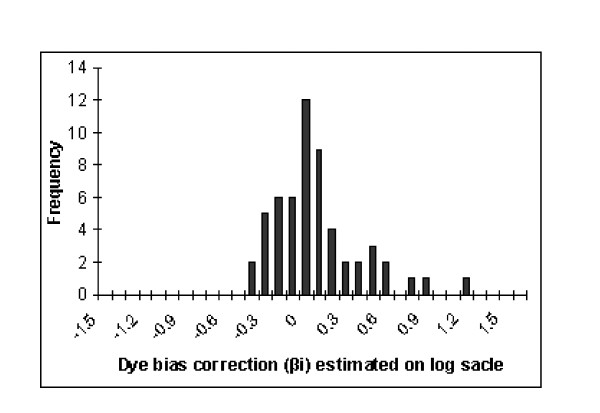
**Histogram of point estimates (median of posterior distribution) of *β*_*i *_for GAPDH**. These point estimates are of the 56 replicates (on the same array) for a house keeping genes (GAPDH) of dataset-2.

Dataset-2 was used to assess the dye-biasness (*β*_*i*_) as it has three replicates of which one has dye orientation reversed. Since the true positives are not known for this dataset, we assessed the dye bias aspect using a housekeeping gene that was replicated 56 times on the array. This is the "Human glyceraldehyde-3-phosphate dehydrogenase (GAPDH, housekeeping gene)", which is assumed to be expressed at a relatively constant level across many different conditions. As a result, the difference *D*_*i *_= *T*_*i*1 _-*T*_*i*2_, *i *= 1,.....,56, between the two conditions for GAPDH should be near zero. Figure 4 displays histograms plotted using the point estimates (median of the posterior distribution) of *D*_*i *_= *T*_*i*1 _-T_*i*2_, *i *= 1,.....,56, obtained from the model. This histogram is centered around zero (as expected) and the non-zero point estimates (median of the posterior distribution) of *β*_*i*_, i = 1, 2,..., 56, for the replicated gene GAPDH indicating the presence of dye-bias (see Figure 5).

### Availability and requirements

Project name: Bayesian Integrated analysis

Availability: Model code (Additional file 5), sample data (Additional file 6), initial conditions (Additional file 7)

Operating system(s): Platform independent

Programming language: WinBUGS

License: Code is freely available for usage and modifications; however, appropriate reference of this article is essential.

## Conclusions

Our focus has been on modeling differential gene expression between two experimental conditions, by proposing an integrated statistical solution where signal correction, systematic array and dye effects, and differential expression, were all modeled jointly. All processing steps were integrated into a common statistically coherent framework, allowing all components and their associated errors to be considered simultaneously. The inference was based on the full posterior distribution of gene expression indices and of their derived quantities, such as difference (*D*_*i*_), rather than on point estimates. In this respect, our approach differs in a fundamental way from most alternative methods which have been proposed in the literature and are build on the idea of statistical significance testing.

The key advantages of the proposed integrated analysis are: (i) robustness of final results towards small variations in outcomes of intermediate steps of the analysis, and (ii) straightforward interpretability of results, when stated in terms of the posterior distributions of differences between the true expression levels obtained under different experimental conditions.

The Bayesian hierarchical models considered here are a step towards a complete integrated approach to the analysis of gene expression data. In future, the model presented here can be extended to include other common steps in the analysis, such as background correction, quality inspection, functional annotation, and clustering. Simultaneous consideration of such additional steps can be expected to lead to further improvements in the estimates and thus to more reliable inferences.

The current model was successfully implemented using WinBUGS software. WinBUGS provides a user-friendly and easily modifiable implementation of Bayesian hierarchical models. This ease of handling and modifying complicated models is balanced by the running time when dealing with large genomic application data. All future extensions (say, incorporating background correction) need to be implemented in C or C++ for a realistic running time of the models. However, comparison of multiple conditions using the integrated model in BUGS (described in here) can be easily speeded up by running the same model with different conditions on different machines.

The results we have obtained from the RhoG experiments are very interesting and provide us several interesting candidate genes for further studies. Many of the genes identified suggest novel links with the cellular machinery.

## Authors' contributions

RG was responsible for model construction, implementation, functional analysis and paper writing. DG helped in the functional analysis and comparison study. PA provided the data and validated the results. EA provided valuable insights in the model construction and helped in paper writing. All authors have read and approved the final manuscript.

## Supplementary Material

Additional file 1 **Dataset-1**. The file (excel) contain pre-processed gene expression intensities (on log scale) for two conditions, each replicated twice, and each replicate scanned three times. The file also contains the name of the genes and their id's. Click here for file

Additional file 2 **Dataset-2**. The file (excel) contain pre-processed gene expression intensities (on log scale) for two conditions, each replicated thrice, and each replicate scanned three times. The file also contains the name of the genes and their id's. Click here for file

Additional file 3 **Details about RNA extraction, probes labeling, and microarray hybridization and qPCR details for the dataset-1 and dataset-2**. The file (word document) contains technical details about how RNA was extracted, probes labeled and finally hybridization carried out for both dataset-1 and dataset-2. The file also explains how qPCR was performed and some qPCR results are listed. Click here for file

Additional file 4 **List of GO terms associated with the differentially expressed genes**. The file (excel) contains the all the GO terms associated with the 270 genes identified as differentially expressed. These GO categories were identified using the DAVID annotation tool. Click here for file

Additional file 5 **Model description**. The file (text) contains the model written in BUGS language. Click here for file

Additional file 6 **Sample data**. The file (text) contains a small dataset demonstrating how the data should be written. Click here for file

Additional file 7 **Initial conditions**. The file (text) specifies the initial conditions that need to be specified for completing model specifications. Click here for file
