# Supplementary material for: Bayesian integrated modeling of expression data: a case study on RhoG
Source: BMC Bioinformatics. 2010 Jun 1;11:295. doi: 10.1186/1471-2105-11-295 (PMC2894040; doi:10.1186/1471-2105-11-295)
Supplement: Additional file 3 — Details about RNA extraction, probes labeling, and microarray hybridization and qPCR details for the dataset-1 and dataset-2. The file (word document) contains technical details about how RNA was extracted, probes labeled and finally hybridization carried out for both dataset-1 and dataset-2. The file also explains how qPCR was performed and some qPCR results are listed. [file 1471-2105-11-295-S3.DOC]

Sample preparation, probe labeling and hybridization for dataset-1

In order to obtain the regulated expression of dominant negative mutant RhoG in Hela cell, RhoG17 cDNA was cloned into pcDNA4/TO vector for expression. Two petri dishes/construct (10 cm each) of T-Rex-HeLa cells (Invitrogen) were transfected with Fugene (Roche) pcDNA4/TO vector or vector with containing RhoG17 cDNA insert. The next day the selection medium (DMEM supplemented with 10% FCS, Blasticidin (2.5 µg/ml) and Zeocin (150 µg/ml)) were added to the plates.

After 12 weeks, clones (hundreds) on the plates were trypsinated and re-plated to form a population of PC control (called PC) and RhoG17 cells. These populations were used in the experiments. Tetracycline (5 µg/ml) was added to plates for induction of expression and after 24 hours samples were collected. In the experiments, DMEM was used without Blasticidin and Zeocin. We repeated this experiment three times.

Total RNA was isolated from confluent cell cultures (RNA easy, Qiagen). RNA amount and quality was checked with UV spectrophotometer and Agilent 2100 Bioanalyzer respectively. Samples (1000 ng/sample) were labeled indirectly using the T7 amplification method (Amino Allyl MessageAmp^TM^ II aRNA Amplification Kit, Ambion) according to manufacturer’s instructions. aRNA (5 µg/sample) was labeled using monoreactive Cy3 and Cy5 dyes (Amersham) followed by purification according the manufacturers instructions. Labeled aRNAs were hybridized on to Agilent Human 4x44K human slides according to the manufacturer’s instructions. Slides were washed according to the instructions and scanned with Axon GenePix 4200 AL (Axon) using alternate PMT settings.

Sample preparation, probe labeling and hybridization for dataset-2

RhoG G12V was fused to the strep tag sequence by standard PCR methods followed by sequence verification. This strep tag-RhoG G12V construct was transferred to a pCI-Neo expression vector (Promega). Control cells were produced by transfecting pCI-Neo vector and the cells expressing RhoG by transfecting pCI-Neo vector with strep tag-RhoG G12V. Both were transfected using Fugene (Roche). Selection medium (DMEM supplemented 400 µg/ml Geneticin) was added to the plates next day. After three weeks of selection, hundreds of clones on plates were trypsinated and re-plated to form a population of RhoG expressing cells and control cell population with pCI-Neo vector only. These populations were used in the experiments.

Control cells (pCI-Neo transfected) and sample cells (RhoG G12Vtransfected) were separately placed in 10 cm petri dishes. The expression of RhoG was enhanced by adding 5 mM butyric acid, which induces expression of genes regulated by the CMV promoter (pCI-Neo). This experiment was repeated twice in different weeks and samples collected after 4 hours. Total RNA was isolated from confluent cell cultures (RNAeasy, Qiagen) and RNA amount and quality was checked by UV spectrophotometer and Agilent 2100 Bioanalyzer respectively.

Total RNA purified from our HeLa cells (25 µg/sample) was labeled directly using Cy3 or Cy5 dCTP (Amersham) in the labeling reaction according to manufacturers instructions (Cyscribe First strand cDNA labeling kit, Amersham). Labeled cDNA was then purified and blocking agents were added (herring sperm DNA (20 µg) (Sigma) Poly-(dA) (20 µg) (Amersham)) followed by drying in vacuum. This probe complex was dissolved in 80 µl of hybridization buffer (50% formamide, (Sigma,) 6xSSC, 0.5% SDS, 5x Denhardt's solution) and incubated at 95ºC for 5 min, cooled to 4ºC and later transferred to ice. The arrays were pre-hybridized at 65ºC in pre-warmed 6xSSC, 0.5% SDS, 1% BSA (Sigma) for 60 minutes. After pre-hybridization the slides were washed three times, 5 minutes each, with milliQ water at room temperature and dried by centrifugation briefly at 2000 rpm. A lifter slip (Erie scientific) was placed onto the spots and samples were added on the slides. The slides were placed in a humid hybridization chamber (Genemachines), which was submerged in water and incubated for 16 hours at 42ºC. The next day microarrays were washed three times, 10 minutes each, in a solution containing 0.1xSSC and 0.1% SDS, and washed another three times, 10 minutes each, in a solution of 0.1xSSC, followed by final three washes with milliQ water and then dried by centrifugation (2000 rpm). Scanning was performed on Scan Array 5000 using alternating laser settings.

**QPCR**

QPCR was performed on ABIprism 7000 machine (Applied Biosystems) in triplicates. Beta-actin was used for normalization. We used SYBR green chemistry (Absolute SYBR Green ROX Mix, ABgene,) for the detection of the amplified fragments. All primers were purchased Oligomer ltd. cDNAs were synthesized using TaqMan Reverse Transcription Reagents (Applied Biosystems).

Primers for detecting transgene RhoG:

tet F: 5’ CCCGCTGATCAGCCTCGACT 3’

tet R: 5’ GCAAACAACAGATGGCTGGC 3’

Primers for detecting endogenous RhoG:

RhoG F: 5’ TCGCTGCTCTTGGAATTGC 3’

RhoG R: 5’ CTGTGGCTCCCTCATTGGA 3’

Primers for detecting beta-actin:

beta actin F: 5’ CTGGAACGGTGAAGGTGACA 3’

beta actin R: 5’ CGGCCACATTGTGAACTTTG 3’

Primers for detecting chimaerin1:

chimaerin1 F: 5’ TCCAGCATGTTTCACAGAGTAAGC 3’

chimaerin1 R: 5’ CAAGCATCAGAAACCAATGACATAG 3’

**QPCR results**

We found RhoG17 in HeLa cells that expressed RhoG 17 mutant was up regulated (5.04x compared to the control cell line) and consequently also expression of endogenous RhoG was elevated (1.47x). We also tested some regulators of Rho-proteins and observed a 1.51x increase in the expression of Chimaerin 1.
